# In vitro fenestrated endovascular aortic repair deployment and cannulation using an electromagnetically steerable guide wire

**DOI:** 10.1016/j.jvscit.2026.102301

**Published:** 2026-05-06

**Authors:** Christian Zielasek, Sebastian Stark, Vladimir Makaloski, Michel Bosiers, Drosos Kotelis, Silvan Jungi

**Affiliations:** Department of Vascular Surgery, Inselspital, Bern University Hospital, University of Bern, Bern, Switzerland

**Keywords:** Aortic aneurysm, Aortic surgery, Basic surgical research, Endovascular surgery, Endovascular therapy, Fenestrated endovascular aortic repair, Magnetic steering, Radiation protection, Robotic endovascular surgery, Robotics

## Abstract

Precise guidewire navigation is critical in branched and fenestrated endovascular aortic repair, where target vessel cannulation often requires multiple devices. This study assessed the feasibility of electromagnetic guidewire navigation in a four-fenestration fenestrated endovascular aortic repair model. A custom-made endograft was deployed in a three-dimensional silicone thoracoabdominal aneurysm model. Renal and visceral arteries were cannulated using a magnetically steerable guidewire and electromagnetic navigation via femoral access. All four target vessels were successfully cannulated using the magnetic guidewire alone; three were stented without adjunctive devices. Magnetically guided navigation enabled accurate cannulation without steerable sheaths or support catheters, potentially reducing procedural complexity.

Branched or fenestrated endovascular aortic repair (fEVAR) relies on precise guidewire control for successful cannulation of renal and visceral arteries. Multiple wires and auxiliary devices are typically required to connect fenestrations with corresponding arteries via covered bridging stents. This process can be technically challenging, time-consuming, and costly, and contributes to substantial radiation exposure for both the operator and patient.^1^

These challenges are particularly pronounced in more complex endovascular aortic procedures, where steep vessel angulations or tortuous anatomies complicate target vessel access. Conventional adjunctive tools such as preloaded guidewires, endovascular snares, and steerable sheaths have led to decreased cannulation times but come with extra costs.^2^, ^3^, ^4^ In some cases, complex anatomy may even result in failed cannulation, necessitating additional invasive techniques such as brachial or axillary access, which are associated with increased morbidity, including nerve injury, bleeding, and stroke rates of up to 2%.^5^, ^6^, ^7^

Magnetic robotic catheterization has previously demonstrated safety and feasibility across multiple clinical domains including cardiac ablation and neurovascular interventions.^8^^,^^9^ Although mechanical robotic systems have previously been explored, the aim of this study was to investigate the feasibility of electromagnetic navigation using a magnetically steerable guidewire and a mobile electromagnetic field generator for target vessel cannulation and stent deployment in fEVAR within an anatomically realistic in vitro model.^10^^,^^11^

## Methods

### Magnetically steerable guidewire prototype and electromagnetic navigation system

**Fig 1:**
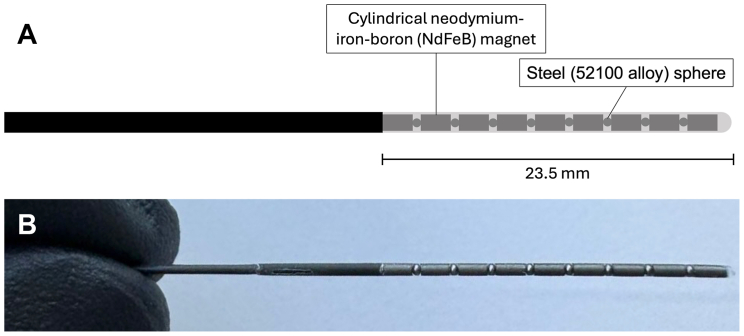
Schematic of the 0.035” magnetically steerable guidewire (MGW) illustrating the segmented magnetic tip **(A)**. Photograph of the MGW **(B)**. *NdFeB*, Neodymium-iron-boron.

The magnetic guidewire and electromagnetic navigation system used in this study have been described in greater detail previously.^12^ Briefly, a commercially available 0.035” stiff guidewire (Glidewire Stiff GS3503, Terumo) was modified with a series of nine cylindrical neodymium-iron-boron (NdFeB) magnets, spaced by steel (52,100 alloy) spheres, to create a magnetically steerable guidewire (MGW). This 23.5-mm–long magnetic tip was encapsulated in a Pebax 35D thermoplastic elastomer jacket to maintain a consistent diameter with the original 0.035” guidewire, ensuring compatibility with standard catheters (Fig 1).

**Fig 2:**
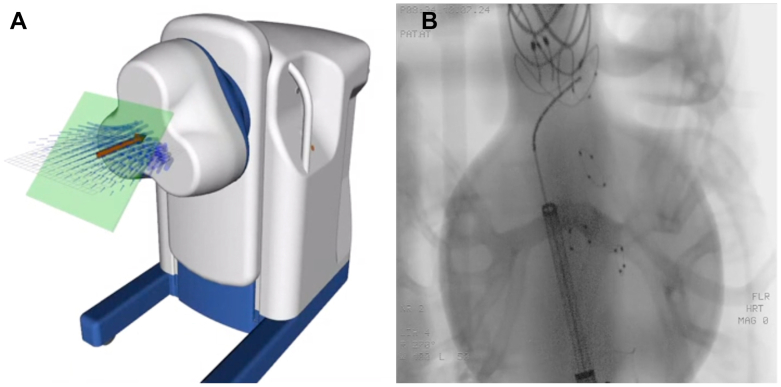
Graphical user interface displaying the field vector **(A)**. Corresponding fluoroscopic image showing the guidewire tip aligned in the selected direction **(B)**.

The electromagnetic navigation system (eMNS) used in this study was developed by the Multi-Scale Robotics Lab at ETH Zurich and is currently being commercialized by Nanoflex Robotics.^13^ It is a mobile, floor-mounted platform (footprint 1200 × 780 mm, height 1400 mm, weight 420 kg), which is positioned next to the patient prior to the procedure depending on the anatomical target (eg, cranial for neurovascular or lateral for abdominal interventions) and remains stationary throughout. The resulting electromagnetic workspace covers the region of interest and does not require repositioning during the procedure. Within the workspace, operators can control the field vector and, therefore, the MGW’s orientation in any direction using an input console linked to a computer, which calculates and adjusts the appropriate currents in the electromagnets (Fig 2). With a peak magnetic field strength of approximately 300 mT at the device surface and substantially lower values within the workspace, the system does not interact with ferromagnetic objects under standard operating room conditions. Despite field strengths well below those of magnetic resonance imaging systems (1.5-3 T), cardiac implantable electronic devices are currently considered a relative contraindication.

### Silicone aortic model

**Fig 3:**
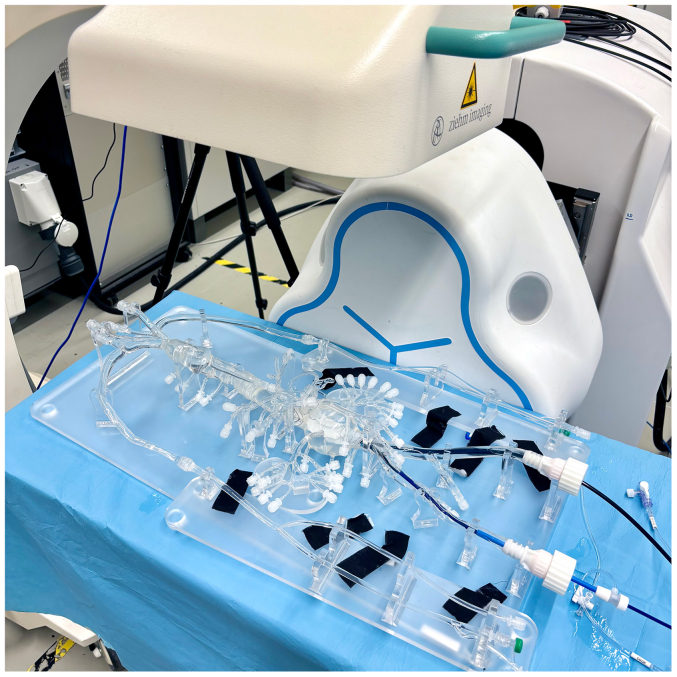
Setup with the three-dimensional (3D) silicone aortic model, the electromagnetic navigation system (eMNS) positioned laterally, and the C-arm cranially, demonstrating spatial arrangement and scale of the system.

The experiment was conducted using a three-dimensional (3D) silicone model of the human aortoiliac anatomy (TrandoMed). The model featured a 60.0 × 65.8 mm paravisceral aortic aneurysm, with an aortic diameter of 19.9 mm at the height of the celiac trunk and 15.5 mm above the aortic bifurcation. The model’s iliac arteries included a relatively straight left iliac artery and a tortuous right iliac artery, with a proximal common iliac diameter of 11.1 mm and a distal external iliac diameter of 7.4 mm. The renal and visceral artery origins measured between 6 mm and 8.7 mm in diameter. The silicone model was filled with soapy water and positioned at a 35° oblique angle on the table to accommodate the limited movement range of the C-arm imposed by the experimental setup. The target area for this experiment, consisting of the juxtarenal aorta and its visceral branches, was fully contained within the workspace of the eMNS and was placed at approximately 220 mm distance to the surface electromagnetic coils. The eMNS remained stationary throughout the entire experimental procedure, and the operators were positioned to the right of the eMNS (Fig 3).

### Fenestrated endovascular aortic repair prosthesis

A custom-made Anaconda Lopro90 abdominal aortic aneurysm fenestrated stent-graft system (CFD3225-080, Terumo Aortic) was selected to fit the dimensions of the silicone model’s paravisceral aneurysm. The endograft featured a total of four fenestrations for the celiac trunk, the superior mesenteric artery, left renal artery, and right renal artery. The position and dimensions of the fenestrations were 0°, 8.0 mm for the celiac trunk; −5°, 9.0 mm for the superior mesenteric artery; 46°, 6.0 mm for the left renal artery; and −70°, 7.0 mm for the right renal artery, respectively.

### Procedure

In the 3D silicone model, access to the left iliac artery was achieved via direct retrograde introduction of a short 6F sheath. The left iliac artery was selected for insertion of the main body due to its relatively straight anatomical structure. Under fluoroscopic guidance using a Vision FD Mobile C-Arm (Ziehm Imaging), a 0.035” MGW was introduced and placed in the aortic arch. Following this, the 6F sheath was exchanged for the fEVAR main body with a 22F outer diameter.

The fEVAR main body was then positioned and deployed, aligning its fenestrations precisely with the renal and visceral artery origins. For cannulation of the fenestrations and its corresponding arteries, a 8F Flexor Ansel Guiding Sheath with a multipurpose tip configuration (G47701, Cook Medical) was inserted to gain access to the more tortuous right iliac artery, followed by the MGW. Using the eMNS controller, precise adjustments were made to remotely steer the MGW’s tip into the renal and visceral artery origins. Tip orientation was controlled via the steering console and visualized both fluoroscopically and on a graphical user interface displaying the direction of the magnetic field (Supplementary Video 1, online only). Once a stable position was achieved within the target arteries, the nonsteerable 8F guiding sheath was advanced using a guiding catheter instead of the dilator due to limited support from the MGW in its current design, and covered bridging stents (BeGraft, Bentley InnoMed) were deployed using the MGW for support (Supplementary Video 2, online only). Fluoroscopic imaging confirmed the correct positioning and patency of all devices following the procedure. Because the Anaconda stent graft features fabric-only reinforced fenestrations without stent rings at the renovisceral level, we additionally tested a Zenith Fenestrated endograft (Cook Medical); steering performance of the MGW remained unaffected.^14^^,^^15^ The procedure was performed by an experienced endovascular surgeon and a vascular surgery trainee under supervision of robotics engineers.

### Study endpoints

The primary endpoint of the study was technical success, defined as accurate deployment of the fenestrated endograft with correct alignment of its fenestrations to the target vessel ostia and successful cannulation of the corresponding target vessels using the MGW. Secondary endpoints were successful deployment of bridging stents, procedure time and fluoroscopy time for each target vessel, the corresponding radiation dose, the need for adjunctive devices or guidewire exchange, and the feasibility and reliability of MGW control using the eMNS. The occurrence of technical issues during the procedure was also systematically recorded.

## Results

**Fig 4:**
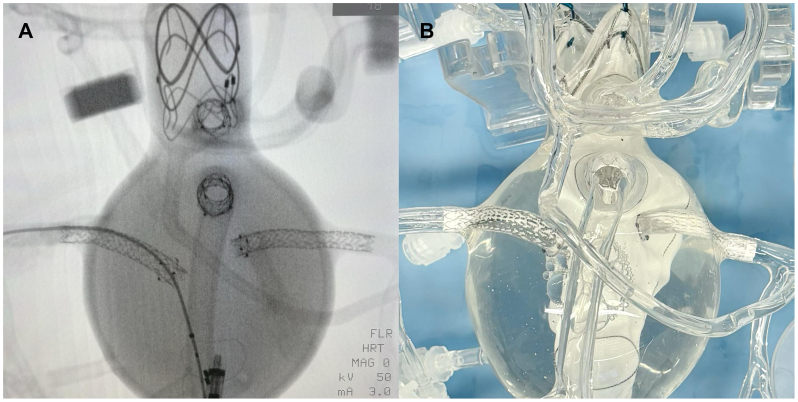
Three-dimensional (3D) silicone aortic model with fenestrated endovascular aortic repair (fEVAR), showing four deployed covered bridging stents under fluoroscopy **(A)** and in a photograph **(B)**.

**Fig 5:**
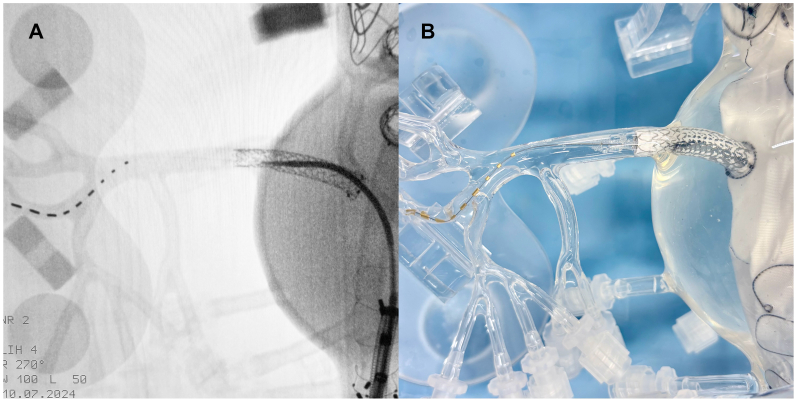
Three-dimensional (3D) silicone aortic model after fenestrated endovascular aortic repair (fEVAR), showing the bridging stent and magnetically steerable guidewire (MGW) positioned in the right renal artery, visualized under fluoroscopy **(A)** and in a photograph **(B)**.

The fEVAR main body was successfully deployed in the silicone model with correct alignment of its fenestrations to the target vessel ostia. All four fenestrations and their corresponding target vessels were successfully cannulated solely with the MGW, without the need for a steerable sheath, resulting in a 100% success rate for cannulation. Covered bridging stents were deployed in three of the target vessels using only the MGW for support (Fig 4). For the highly tortuous celiac artery, after successful cannulation, the MGW was exchanged for a stiffer guidewire (Hi-Torque Supracore, Abbott) to enable the placement of a nonsteerable 8F sheath and subsequent deployment of a covered stent. The eMNS operated without technical issues. Notably, there was a considerable gap between the origins of the renal arteries and the corresponding fenestrations (Fig 5). Nevertheless, successful cannulation was achieved.

The fluoroscopy times and corresponding doses for each target artery cannulation were recorded. Fluoroscopy times for target vessel cannulation ranged from approximately 4 to 12 minutes, with corresponding radiation doses between 179.0 and 399.5 cGy cm^2^ (Table).

**Table:** Fluoroscopy time, dose, and technical success rates for cannulation and stent placement in each target artery using only the magnetically steerable guidewire (*MGW*)

Target artery	Fluoroscopy time, minutes:seconds	Fluoroscopy dose, cGycm^2^	Cannulation success, %	Stent placement success, %
Celiac trunk	4:46	225.8	Yes	No^a^
SMA	9:39	399.46	Yes	Yes
LRA	11:56	374.5	Yes	Yes
RRA	6:03	179	Yes	Yes
Total	32:24	1178.76	4/4 (100)	Three-fourths (75)

*LRA*, Left renal artery; *RRA*, right renal artery; *SMA*, superior mesenteric artery.

a In the celiac artery, following successful cannulation, the MGW was exchanged for a stiffer guidewire (Hi-Torque Supracore, Abbott), enabling subsequent stent placement.

## Discussion

Target vessel cannulation remains one of the most time-consuming and technically demanding steps during fEVAR. Conventional techniques often require sequential use of multiple wires, catheters, and auxiliary devices. This procedural complexity may lead to longer procedure times, higher radiation exposure, greater contrast use, extended hospital stays, and increased risk of intraoperative complications.^16^, ^17^, ^18^ The system examined in this study aimed to address these limitations by offering precise guidewire control through electromagnetic steering while maintaining compatibility with standard endovascular devices. In our in vitro model, we successfully performed full deployment of a four-fenestration endograft and cannulated all target vessels exclusively using the MGW, without the need for support catheters, steerable sheaths, or upper extremity access. To our knowledge, this is the first study to successfully evaluate electromagnetic navigation for target vessel cannulation and stent deployment in fEVAR. However, the in vitro model, although designed to approximate human aortic anatomy, does not fully reflect lifelike conditions. Furthermore, the experiment was performed as a single proof-of-concept procedure, precluding assessment of reproducibility.

Robotic catheter navigation systems have been developed to enhance precision and operator safety. Early platforms such as Magellan (Hansen Medical/Auris Surgical Robotics) demonstrated feasibility in aortic repair and reduced radiation exposure, but required large sheath profiles and proprietary catheters.^19^, ^20^, ^21^, ^22^ The CorPath GRX (Corindus Vascular Robotics/Siemens Healthineers) offered improved workflow integration with remote wire and device manipulation from a shielded cockpit, yet remained limited in complex anatomies due to restricted guide catheter control and lack of compatibility with over-the-wire devices.^23^, ^24^, ^25^

eMNSs represent a distinct approach. Systems such as Niobe or its successor Genesis (Stereotaxis) employ externally modulated magnetic fields to steer magnetically responsive devices without pull-wires, eliminating friction and mechanical instability while allowing a high level of precision with full 3D tip control and remote operation from radiation-shielded workstations.^26^ However, they required large fixed magnets, reinforced operating rooms, and major financial investment, which restricted widespread adoption.^27^, ^28^, ^29^ In contrast, the compact coil-based eMNS used in this study (Navion, Nanoflex Robotics) generates and modulates a magnetic field in real time via controlled currents in multiple electromagnetic coils, enabling guidewire steering without mechanical movement of large magnets. Combined with proprietary cooling, this enables a smaller, mobile system without the need for reinforced operating rooms.^30^ It allows wide-angle tip deflection, real-time control, and compatibility with standard tools, although it requires proprietary MGWs.^12,1312^

A common limitation of robotic systems is the lack of haptic feedback.^27^ In contrast, the MGW combines remote magnetic steering with manual wire advancement, preserving tactile sensation. This hybrid control proved advantageous in aneurysm sacs and tortuous vessels.^12^^,^^13^ Nonetheless, the current design of the MGW, particularly its stiffness and the length of the magnetic tip, requires further refinement. During celiac trunk cannulation, the rigid magnetic tip limited flexibility and bending beyond 90°, whereas the abrupt transition to the stiffer shaft caused buckling under forward pressure. Although cannulation was achieved, the wire did not provide adequate support for stent exchange, necessitating replacement with a stiff wire. Although such challenges are not unique to the MGW and would likely occur with conventional guidewires as well, alternative tip designs may improve performance in difficult anatomies.

Technical limitations of our benchtop setup included image artifacts caused by proximity of the detector to the field generator, which can be mitigated by increasing vertical separation between the generator and the imaging system. However, this comes at the cost of higher radiation dose and remains particularly problematic during pronounced lateral angulations. Another limitation was C-arm mobility, restricted by the mounting feet of the eMNS, which are required to support the system’s weight but impose geometric constraints. In addition, the current steering setup is not yet optimized for intuitive single-operator use. Although feasible, handling is most ergonomic when guidewire advancement and tip control are performed by two operators. Despite these challenges, the compact design still represents a substantial improvement over earlier eMNS platforms by reducing infrastructure requirements and setup time, both critical for clinical translation.^28^

Beyond technical feasibility, electromagnetic navigation may decrease overall procedural complexity and minimize perioperative complications by improving cannulation success and further reducing the need for auxiliary access routes.^27^^,^^31^ Early experience suggests that proficiency can be achieved relatively quickly, and robotic systems may even accelerate skill acquisition among beginners.^12^^,^^32^

Importantly, eMNSs also hold promise for radiation dose reduction. By enabling remote navigation from shielded workstations, the occupational dose could be substantially lowered.^12^^,^^27^, ^28^, ^29^ This benefit, well-established in electrophysiology and neurointerventional applications, could have particular relevance for complex aortic procedures, which often involve prolonged fluoroscopy times and high radiation exposure.^33^, ^34^, ^35^, ^36^ Future integration of automated wire advancement may allow fully remote procedures.^9^^,^^37^

Several hurdles remain for widespread adoption, including costs, workflow integration, and the need for prospective trials to establish safety, efficacy, and cost-effectiveness compared with current standards.^27^^,^^38^

## Conclusions

Electromagnetic navigation platforms hold substantial potential to simplify complex aortic interventions, improve procedural safety, and expand access to advanced endovascular therapies. To enhance clinical applicability, future systems may incorporate the electromagnetic navigation platform directly into the C-arm architecture, thereby addressing current integration limitations. Further preclinical in vivo studies will be essential to comprehensively assess system performance, safety, and operator usability prior to clinical translation.

## Funding

This work was funded through departmental resources.

## Disclosures

None.

## Appendix

Supplementary Video 1 Fluoroscopy demonstrating cannulation of the right renal artery using the magnetically steerable guidewire (MGW), with the graphical user interface displaying the corresponding magnetic field vector in the upper left corner. The video is assembled from sequential fluoroscopic recordings obtained during the procedure.

Supplementary Video 2 Fluoroscopy demonstrating superior mesenteric artery cannulation with buckling at the transition zone of the magnetically steerable guidewire (MGW), followed by catheter-assisted sheath advancement and stent deployment. The video is assembled from sequential fluoroscopic recordings obtained during the procedure.
